# The genome of *Paenibacillus sabinae* T27 provides insight into evolution, organization and functional elucidation of *nif* and *nif*-like genes

**DOI:** 10.1186/1471-2164-15-723

**Published:** 2014-08-27

**Authors:** Xinxin Li, Zhiping Deng, Zhanzhi Liu, Yongliang Yan, Tianshu Wang, Jianbo Xie, Min Lin, Qi Cheng, Sanfeng Chen

**Affiliations:** Key Laboratory for Agrobiotechnology, Ministry of Agriculture, China Agricultural University, Beijing, 100193 P. R. China; Key laboratory of Soil Microbiology, Ministry of Agriculture, China Agricultural University, Yuanmingyuan west road no.2, Haidian District, Beijing 100193 P. R. China; Biotechnology Research Institute, Chinese Academy of Agricultural Sciences, Beijing, 100081 P. R. China

**Keywords:** *Paenibacillus sabinae* T27, *nif gene cluster*, *nif*-*like gene*, *Genome sequence*

## Abstract

**Background:**

Most biological nitrogen fixation is catalyzed by the molybdenum nitrogenase. This enzyme is a complex which contains the MoFe protein encoded by *nifDK* and the Fe protein encoded by *nifH*. In addition to *nifHDK*, *nifHDK*-like genes were found in some Archaea and Firmicutes, but their function is unclear.

**Results:**

We sequenced the genome of *Paenibacillus sabinae* T27. A total of 4,793 open reading frames were predicted from its 5.27 Mb genome. The genome of *P. sabinae* T27 contains fifteen nitrogen fixation (*nif*) genes, including three *nifH*, one *nifD*, one *nifK*, four *nifB*, two *nifE*, two *nifN*, one *nifX* and one *nifV*. Of the 15 *nif* genes, eight *nif* genes (*nifB*, *nifH*, *nifD*, *nifK*, *nifE*, *nifN*, *nifX* and *nifV*) and two non-*nif* genes (*orf1* and *hesA*) form a complete *nif* gene cluster. In addition to the *nif* genes, there are nitrogenase-like genes, including two *nifH*-like genes and five pairs of *nifDK*-like genes. IS elements on the flanking regions of *nif* and *nif*-like genes imply that these genes might have been obtained by horizontal gene transfer. Phylogenies of the concatenated 8 *nif* gene (*nifB*, *nifH*, *nifD*, *nifK*, *nifE*, *nifN*, *nifX* and *nifV*) products suggest that *P. sabinae* T27 is closely related to *Frankia*. RT-PCR analysis showed that the complete *nif* gene cluster is organized as an operon. We demonstrated that the complete *nif* gene cluster under the control of σ^70^-dependent promoter enabled *Escherichia coli* JM109 to fix nitrogen. Also, here for the first time we demonstrated that unlike *nif* genes, the transcriptions of *nifHDK*-like genes were not regulated by ammonium and oxygen, and *nifH*-like or *nifD*-like gene could not restore the nitrogenase activity of *Klebsiella pneumonia nifH*^−^ and *nifD*^−^ mutant strains, respectively, suggesting that *nifHDK*-like genes were not involved in nitrogen fixation.

**Conclusions:**

Our data and analysis reveal the contents and distribution of *nif* and *nif*-like genes and contribute to the study of evolutionary history of nitrogen fixation in *Paenibacillus*. For the first time we demonstrated that the transcriptions of *nifHDK*-like genes were not regulated by ammonium and oxygen and *nifHDK*-like genes were not involved in nitrogen fixation.

**Electronic supplementary material:**

The online version of this article (doi:10.1186/1471-2164-15-723) contains supplementary material, which is available to authorized users.

## Background

Biological nitrogen fixation, the conversion of atmospheric N_2_ to NH_3_, plays an important role in the global nitrogen cycle and in world agriculture [1]. Most biological nitrogen fixation is catalyzed by the molybdenum nitrogenase. This enzyme is a complex which contains the MoFe protein encoded by *nifDK* and the Fe protein encoded by *nifH*. The MoFe protein contains two metalloclusters: FeMo-co, a [Mo-7Fe-9S-C-homocitrate] cluster which serves as the active site of substrate binding and reduction and the P-cluster, a [8Fe-7S] cluster which shuttles electrons to FeMo-co [2, 3]. Previous biochemical and genetic studies on *Klebsiella pneumoniae* carrying twenty *nif* genes on 24-kb region genes and *Azotobacter vinelandii* revealed that *nifH*, *nifD*, *nifK*, *nifE*, *nifN*, *nifX nifB*, *nifQ*, *nifV*, *nifY*, *nifU nifS*, *nif*Z and *nifM* contribute to the synthesis and maturation of nitrogenase [2, 3].

Contents and organization of *nif* genes varied significantly among N_2_-fixing organisms. For example, in *K. pneumoniae*, twenty *nif* genes are co-located within a ~24 kb cluster [4], whereas in *A. vinelandii* the *nif* genes are more dispersed and distributed as two clusters in genome [5]. There is usually only one *nifH* gene and the *nifH*, *nifD* and *nifK* genes are transcribed as a single unit in many diazotrophs, such as *K. pneumoniae* and *A. vinelandii*. However, multiple *nifH* genes were found in a few diazotrophs. For examples, *Rhizobium leguminosarum* bv. *phaseoli* possesses three *nifH* genes [6] and *Clostridium pasteurianum* W5 has six *nifH* homologs [7].

Nitrogen fixation is sporadically distributed among prokaryote families: Proteobacteria, Firmicutes, Archaea, Cyanobacteria and Actinobacteria [8]. The incomplete distribution pattern and the difference in contents and organization of *nif* genes raise the question of origins and evolution of Mo-nitrogenase. Two conflicting hypotheses for the origin of Mo-nitrogenase have been proposed on the basis of phylogenetic examination of Mo-nitrogenase protein sequences (NifHDK) [9]. The last common ancestor (LCA) hypothesis implies that the Mo-nitrogenase had its origin in a common ancestor of the bacterial and archaeal domains. According to the LCA model gene loss has been extensive and accounts for the fact that nitrogenase is found neither in eukaryotes nor in many entire phyla of prokaryotes. The Methanogen origin hypothesis implies that nitrogen fixation originated from methanogenic archaea and subsequently was transferred into a primitive bacterium via lateral gene transfer. Recent studies based on phylogenetic analysis of NifHDK sequences supported the Methanogen origin hypothesis and implied that Mo-nitrogenase evolved in the anaerobic and hydrogenotrophic methanogens with acquisition in the bacterial domain via lateral gene transfer involving an anaerobic member of the Firmicutes [10].

Firmicutes have been thought to play an important role in evolution of nitrogen fixation. Studies on evolution of nitrogen fixation in Firmicutes mainly focused on the anaerobic diazotrophic *Clostridia*. Although *Paenibacillus* is a genus of Firmicute, its nitrogen fixation traits and evolution remains unclear. It is well known that *Paenibacillus* is a genus of Gram-positive, facultative anaerobic, endospore-forming bacteria, originally included within the genus *Bacillus* and then reclassified as a separate genus in 1993 [11]. Bacteria belonging to this genus have been detected in a variety of environments such as soil, water, rhizosphere, vegetable matter, forage and insect larvae, as well as clinical samples [12]. Nitrogen-fixing *Paenibacillus* species have great potential for use as a bacterial fertilizer in agriculture, but genomic information of these bacteria is lacking.

Here we report the complete genome sequence of *P. sabinae* T27 which is a nitrogen-fixer isolated from the rhizosphere of plant *Sabina squamata* by our laboratory [13]. The whole genome analysis not only reveals the organization and distribution of nitrogen-fixing genes and nitrogenase-like genes, but also provides insight into the evolution of *nif* genes in *Paenibacillus*. Furthermore, we demonstrate that the complete *nif* gene cluster consisting of ten genes (*nifB*, *nifH*, *nifD*, *nifK*, *nifE*, *nifN*, *nifX*, *orf1*, *hesA* and *nifV*) of *P. sabinae* T27 is a functional unit for nitrogen fixation. Here for the first time we demonstrated that *nifHDK*-like genes are not involved in nitrogen fixation.

## Results and discussion

### General features of *Paenibacillus sabinae*T27 genome

The complete genome of *P. sabinae* T27 is composed of a single circular molecule of 5,270,569 base pairs (bp) with an average G + C content of 52.64%. The circular chromosome has a total of 4,849 putative protein-coding sequences (CDS), 26 rRNAs (8 copies of 16S-23S-5S operons and 1 copy of 16S-23S operon) and 82 tRNAs (Table 1). Among the predicted genes, 3,538 were assigned putative functions, covering 72.96% of the genome, and 1,311 encoded hypothetical proteins (Table 1). Twenty eight insertion sequence (IS) elements were identified in the *P. sabinae* T27 genome.

**Table 1:** **General features of the genome of** ***P. sabinae*** **T27**

Attribute	Value
Complete genome size, bp	5, 270, 569
G + C%	52.64
Protein-coding sequences	4,849
Genes with assigned function	3,538
Genes with unknown function	1,311
Average CDs size	941
Percent of coding region%	72.96
No. of rRNAs	26
No. of tRNAs	82
Insertion sequence (IS) elements	28

### Comparative genomics of *P. sabinae*T27

**Figure 1:**
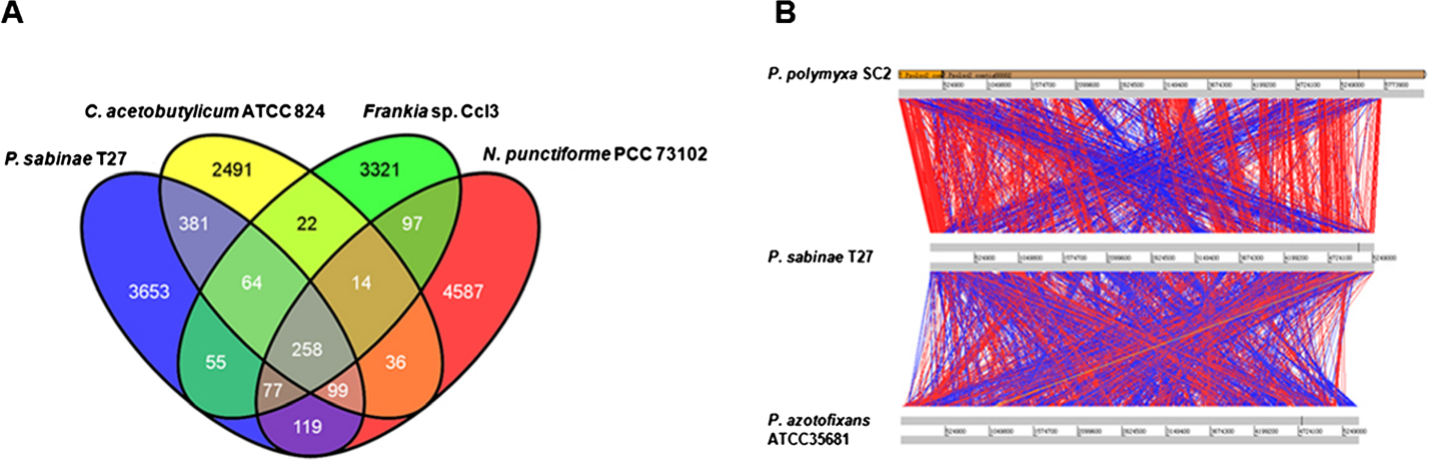
**Comparative analysis of the complete genome sequence of** ***P. sabinae*** **T27. (A)** Alignment of the chromosomes from *P. sabinae* T27, *P. azotofixans* ATCC35681 and *P. polymyxa* SC2, generated using the Artemis Comparison Tool. The gray bands located at the top, middle and bottom represent the forward and reverse DNA strands for the chromosome sequences. The red lines correspond to regions of similarity between two chromosomes. The blue lines correspond to regions of rearrangement and recombination between two chromosomes. White regions are those that are unique to one strain. **(B)** Venn diagram depicting the shared genes which were present in all the *P. sabinae* T27, *C. acetobutylicum* ATCC 824, *Frankia* sp. CcI3 and *N. punctiforme* PCC 73102 and the specific genes which were present and absent from all the four species, and vice-versa.

Previous phylogeny based on *nifHDK* showed that Firmicutes, cyanobacteria and actinobacteria are closely related [10]. Here we compared the genomes of *P. sabinae* T27, *Clostridium acetobutylicum* ATCC 824 (a member of Firmicutes), *Frankia* sp. CcI3 (an actinobacterium) and *Nostoc punctiforme* PCC 73102 (a cyanobacterium). The four species had the core genome of 258 putative protein-coding genes (Figure 1A). There are 802 genes which are shared by *P. sabinae* T27and *C. acetobutylicum* ATCC 824, there are 454 genes which are shared by *P. sabinae* T27and *Frankia* sp. CcI3, and there are 553 genes which are shared by *P. sabinae* T27 and *N. punctiforme* PCC 73102. The shared genes by *P. sabinae* T27and *C. acetobutylicum* are more than those shared by *P. sabinae* T27with *Frankia* sp. CcI3 or *N. punctiforme*. The results are consistent with the fact that *Paenibacillus* is more closely related to *Clostridium* than to *Frankia* and cyanobacteria, since *Paenibacillus* and *Clostridium* belong to the same Firmicutes. Furthermore, the genome of *P. sabinae* T27 was compared with those of the closely related *Paenibacillus azotofixans* ATCC35681 (a nitrogen-fixer) [14] and Paenibacillu polymyxa SC2 (a non-nitrogen-fixer) [15] (Figure 1B). Genome sizes of P. sabinae T27, P. azotofixans ATCC35681 and *P. polymyxa* SC2 are 5.27 Mb, 5.44 Mb and 6.24 Mb, respectively. Chromosome alignments showed higher level of conservation of genome architecture between *P. sabinae* T27 and *P. polymyxa* SC2 than that between *P. sabinae* T27 and *P. azotofixans* ATCC35681.

### Central metabolism

*P. sabinae* T27 is a nitrogen-fixing bacterium isolated from the rhizosphere of the plant *Sabina squamata*[13]. The bacterium contains a wide spectrum of genes for carbon utilization and carbohydrate, amino acid and inorganic ion transport. The genome of *P. sabinae* T27 contains the complete set of genes for the pentose phosphate pathway (PPP) (Additional file 1: Figure S1). In addition to the metabolism of pentose, the non-oxidative PPP allows the production of intermediates necessary for nucleic acid synthesis. It contains the complete set of genes for the glycolysis pathway and allows production of acetyl-CoA. In the presence of external electron acceptors, acetyl-CoA may be completely oxidized via the citrate cycle (TCA cycle), which is encoded by the *P. sabinae* T27 genome (Additional file 1: Figure S1). Although the gene coding for the classical malate dehydrogenase (MDH1, EC:1.1.1.37) in TCA cycle is absent, another malate dehydrogenase (MQO, EC:1.1.5.4) gene which might be involved in pyruvate metabolism pathway metabolizing oxaloacetate to malate, is found in the genome of *P. sabinae* T27.

Sucrose is the common carbon source used for isolation of *P. sabinae* T27 [13]. The genome of the bacterium has the sucrose-6-phosphate hydrolase and alpha-glucosidase for metabolizing sucrose to glucose and fructose. Transporter systems are an important element for bacteria to communicate with their environment. The genome of *P. sabinae* T27 contains an extensive set of 247 transport related genes. Of the 247 transport related genes, 64 are involved in carbohydrate transport, 66 encode components of amino acid transporters and 107 encode components of inorganic ion transporters. Importantly, Fe (iron), molybdenum, sulfate and NH_4_^+^ are related to nitrogen fixation and nitrogen metabolism.

### Nitrogen fixation and nitrogenase-like genes

**Figure 2:**
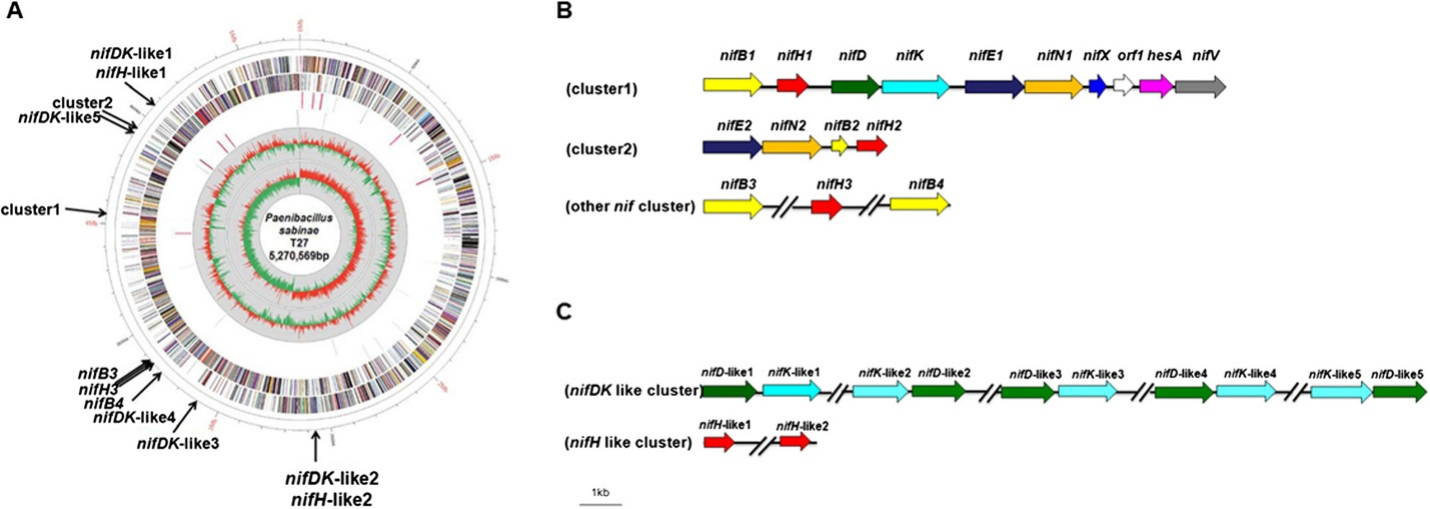
**Chromosome map and distribution of** ***nif*** **genes in** ***P*** **.** ***sabinae*** **T27. A.** Chromosome map. (From the outer to the inner concentric circle) Circles 1 and 2 are genes encoded by leading and lagging strands, respectively. Coding sequences are color-coded by COG categories. Circle 3 and 4 are distribution of tRNA (black) genes and rRNA (red) genes in the + strand and – strand, respectively. Circle 5 and 6 show G + C content and GC skew (G-C/G + C), respectively. The positions of *nif* genes and *nif* clusters are indicated in the chromosome map. **B.** The organization of *nif* genes. **C.** The organization of *nif*-like genes.

One of the most distinct features of *P. sabinae* T27 is its ability to fix nitrogen. The genome of *P. sabinae* T27 contains fifteen *nif* genes, including four *nifB*, three *nifH*, one *nifD*, one *nifK*, two *nifE*, two *nifN*, one *nifX* and one *nifV*. Of the 15 *nif* genes, eight *nif* genes (*nifB*, *nifH*, *nifD*, *nifK*, *nifE*, *nifN*, *nifX* and *nifV*) and two non-*nif* genes (*orf1* and *hesA*) which are located between *nifX* and *nifV* form a complete *nif* gene cluster, the four *nif* genes (*nifE*, *nifN*, *nifB* and *nifH*) are clustered together and the other three *nif* genes (two *nifB* and one *nifH*) are scattered at different locations (Figure 2). In addition to the *nif* genes, there are nitrogenase-like genes, including two *nifH*-like and five pairs of *nifDK*-like genes. Our results are consistent with the reports that *nifHDK*-like genes existed in Archaea and Firmicutes [8]. Interestingly, genome of *P. sabinae* T27 does not contain transcription regulatory gene *nifA* which is found in almost all of Gram-negative diazotrophs, such as in *K. pneumoniae*, *A. vinelandii* and *Pseudomonas stutzeri* A1501 [16]. The lack of *nifA* suggests that there may be a different regulation mechanism of nitrogen fixation in *P. sabinae* T27.

### The content and organization of the complete *nif*gene cluster

Bioinformatics analysis revealed that the ten genes *nifBHDKENXorf1hesAnifV* within the complete *nif* gene cluster are organized as an operon within an 11 kb region. The gene designated as *hesA* is also found in *Frankia*[17] and cyanobacteria [18]. The *orf1*, whose predicted product is a hypothetical protein, is also found in several N_2_-fixing *Paenibacillus* species [19]. The predicted product of HesA shares ~ 45% identity with the putative molybdenum cofactor biosynthesis protein HesA. HesA is a member of the ThiF-MoeB-HesA family and contains an N-terminal nucleotide binding domain and a C-terminal MoeZ/MoeB-like domain. The gene content and organization of the complete *nif* gene cluster is unique to *Paenibacillus*[19, 20]. Although *Paenibacillus* and *Clostridium* are the members of the Firmicute, their *nif* gene content and organization varied greatly. For example, *nifN*-*B* fusion gene was found in the *nif* gene clusters of the three species of *Clostridia*: *C. acetobutylicum*, *C. beijerinckii*, and *C. pasteurianum*. Also, there are two genes *nifI1* and *nifI2* located between *nifH and nifDK in C. acetobutylicum* and *C. beijerinckii*[21]. Previous studies demonstrated that *nifI1* and *nifI2* are not essential for nitrogen fixation, but serve a regulatory function [22]. Actually, the *nif* gene content and organization of *Clostridium* spp. are more similar to those of *Methanosarcina acetovorans* and *Methanococcus maripaudis*, since two genes *nifI1* and *nifI2* also exist between *nifH* and *nifDK* in these archaea.

### IS may play important roles in the evolution of the *nif*and *nif*-like genes

**Figure 3:**
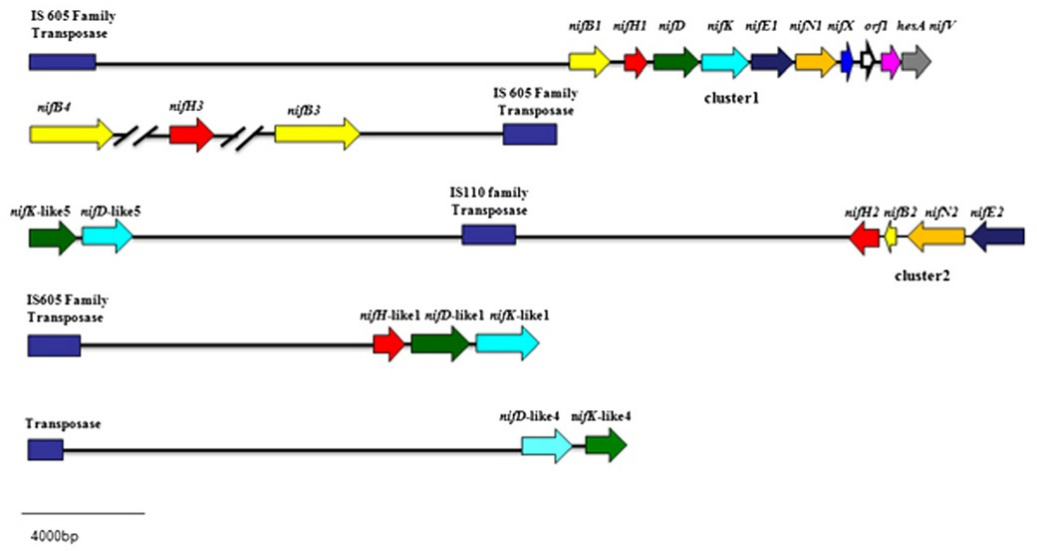
***nif*** **and** ***nif*** **-like genes linked to transposonal elements.**

As described above, twenty eight insertion sequence (IS) elements, belonging to six transposase families were identified in *P. sabinae* T27 chromosome. IS elements were found to be located on the flanking region of the complete *nif* gene cluster, other *nif* genes and *nif*-like genes (Figure 3). It is generally accepted that IS abundance correlates positively with the frequency of horizontal gene transfer (HGT) [23]. IS elements can mediate the transfer of genetic information (such as antibiotic resistance and new metabolic capabilities) between genomes or between replicons of the same genome and they can also induce duplications, deletions, and rearrangements of genetic information [24]. The existence of transposase in the flanking region of the complete *nif* gene cluster suggests that the *nif* cluster might be acquired in *P. sabinae* T27 by HGT event from other diazotrophs and the additional *nifBHEN* genes and *nifHDK*-like genes might be horizontally transferred or duplicated. The *nif* genes acquired by HGT were also reported in several diazotrophs. For example, A sequence reminiscent of a transposase gene located just upstream the *nif* cluster in *Herbaspirillum seropedicae* is an indicative of HGT event [25]. It was generally recognized that variations of G + C contents between *nif* cluster and genome are indicative of HGT. For example, G + C content of the *nif* gene cluster was higher than the average of the entire genome (66.8% vs. 63.8%) in *P. stutzeri* A1501 [16]. However, we found that the G + C contents of the complete *nif* gene cluster of *P. sabinae* T27 is as same as the average of the entire genome (52.64% vs. 52.63%), suggesting that the complete *nif* gene cluster of *P. sabinae* T27 has undergone a longer time of evolution.

### Evolution of *nif*and *nif*-like genes of *P. sabinae*T27

**Figure 4:**
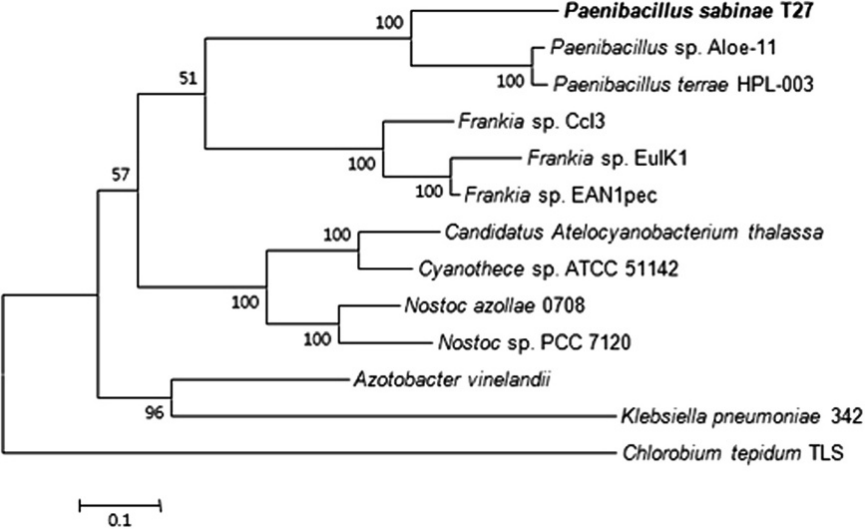
**Maximum-likelihood tree based on NifBHDKENXV protein sequences of** ***P. sabinae*** **T27 and the representative microorganisms.** The numbers at the nodes indicate levels of bootstrap support (%) based on a neighbor-joining analysis of 100 resampled datasets; only values at or above 50% are given, Bar 0.1 substitutions per amino acid position.

To further evaluate the evolution of nitrogen fixation in *P. sabinae* T27, we reconstructed the phylogenies based on the concatenation of the NifBHDKENXV sequences (Figure 4). Notably, the Nif protein sequences of *P. sabinae* T27 used for the phylogenetic trees were from the complete *nif* cluster. The phylogenetic tree showed that *Paenibacillus* and *Frankia* are sister groups, suggesting that *P. sabinae* T27 may originate from a common ancestor with *Frankia*. Also, we constructed the phylogenetic trees based on the HesA and Orf1 sequences which are contained within the complete *nif* gene cluster. The HesA phylogenetic tree revealed supported that *Paenibacillus* and *Frankia* are sister groups (Additional file 2: Figure S5). The Orf1 phylogenetic tree showed that *P. sabinae* T27 is closely related to *Clostridium* (Additional file 3: Figure S6). IS element on the flanking region of the complete *nif* cluster suggested that the complete *nif* cluster may have been acquired in *P. sabinae* T27 by HGT. Interestingly, these data revealed that although *Paenibacillus* and *Clostridium* are the members of the Firmicutes, their *nif* genes are not very closely related.

**Figure 5:**
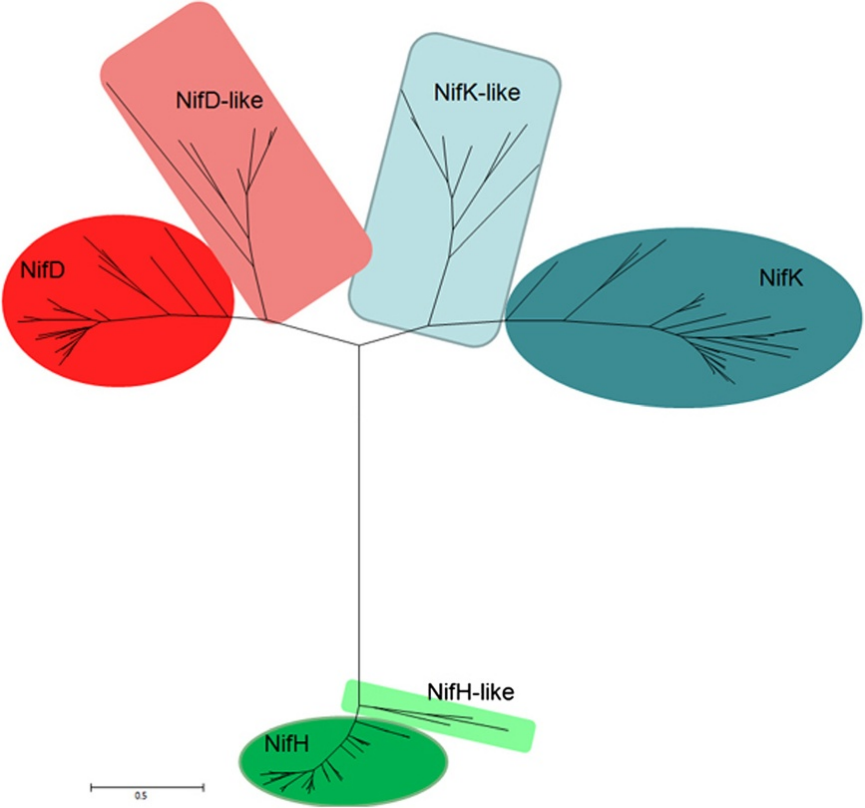
**Maximum-likelihood phylogenetic tree of NifHDK/NifHDK-like sequences.** NifHDK/NifHDK-like sequences were derived from *P. sabinae* T27 and the representative microorganisms. The numbers at the nodes indicate levels of bootstrap support (%) based on a neighbor-joining analysis of 100 resampled datasets; only values at or above 50% are given, Bar 0.1 substitutions per amino acid position.

The complete genome sequence revealed that there are three *nifH*, one *nifD*, two *nifH*-*like*, five pairs of *nifDK*-like genes in *P. sabinae* T27. Here we constructed phylogenetic trees with real NifH/NifD/NifK and NifH/NifD/NifK-like sequences (Figure 5) and the phylogenetic tree revealed that NifH/NifD/NifK-like sequences are clearly divergent from conventional nitrogenase. All NifH-like, NifD-like and NifK-like sequences are clustered together by themselves, suggesting that they may have been resulted from duplication.

As described above, in addition to the ten genes *nifBHDKENXorf1hesAnifV* within the complete *nif* gene cluster, three *nifB*, two *nifH*, one *nifE* and one *nifN* genes exist in the genome of *P. sabinae* T27. Here we further constructed NifB, NifH and the concatenated NifEN phylogenetic trees (Additional files 4, 5, 6: Figures S2-S4) and phylogenetic analysis revealed that these multiple *nifB*, *nifH* and *nifEN* are clustered with their own corresponding genes within the complete *nif* gene cluster, suggesting that they may result from duplication of *nifB*, *nifH*, *nifE* and *nifN*, respectively, of the complete *nif* gene cluster.

### Characterization of multiple nitrogenase-like genes

**Figure 6:**
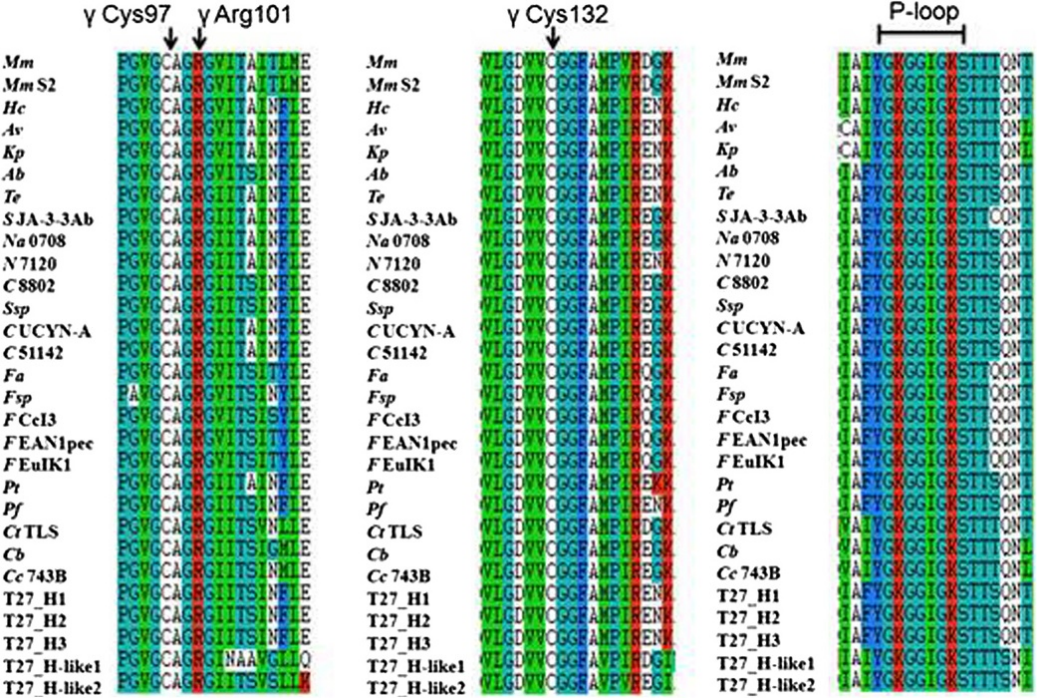
**Alignment surrounding the MgATP binding motif (bar) and 4Fe-4S coordinating cysteines (vertical arrows) for NifH and NifH-like protein sequences from** ***P. sabinae*** **T27. Numbering is based on** ***A. vinelandii*** **NifH.** *Ab* = *A. brasilense*, *Av* = *A. vinelandii*, *Ct* TLS = *C. tepidum* TLS, *Cb* = *Clostridium beijerinckii*, *Cc* 743B = *C. cellulovorans* 743B, *C* UCYN-A = *Cyanobacterium* UCYN-A, *C* 51142 = *C*. sp. ATCC 51142, *C* 8802 = *C. sp*. PCC 8802, *Fal* = *F. alni* ACN14a, *Fsp* = *Frankia* sp., *F* CcI3 = *F*. sp. CcI3, *F* EAN1pec = *F*. sp. EAN1pec, *F* EuIK1 = *F. sp*. EuIK1, *Hc* = *H.chlorum*, *Kp* = *K. pneumoniae* 342, *Mm* = *M. maripaludis*, *Mm* S2 = *M. maripaludis* S2, *Na* 0708 = *N. azollae* 0708, *N* 7120 = *N*. sp. PCC 7120, *Pf* = *P. fujiensis*, *Pt* = *P. terrae* HPL-003, *Ssp* = *S*. sp., *S* JA-3-3Ab = *S*. sp. JA-3-3Ab, *Te* = *T. erythraeum* IMS101.

**Figure 7:**
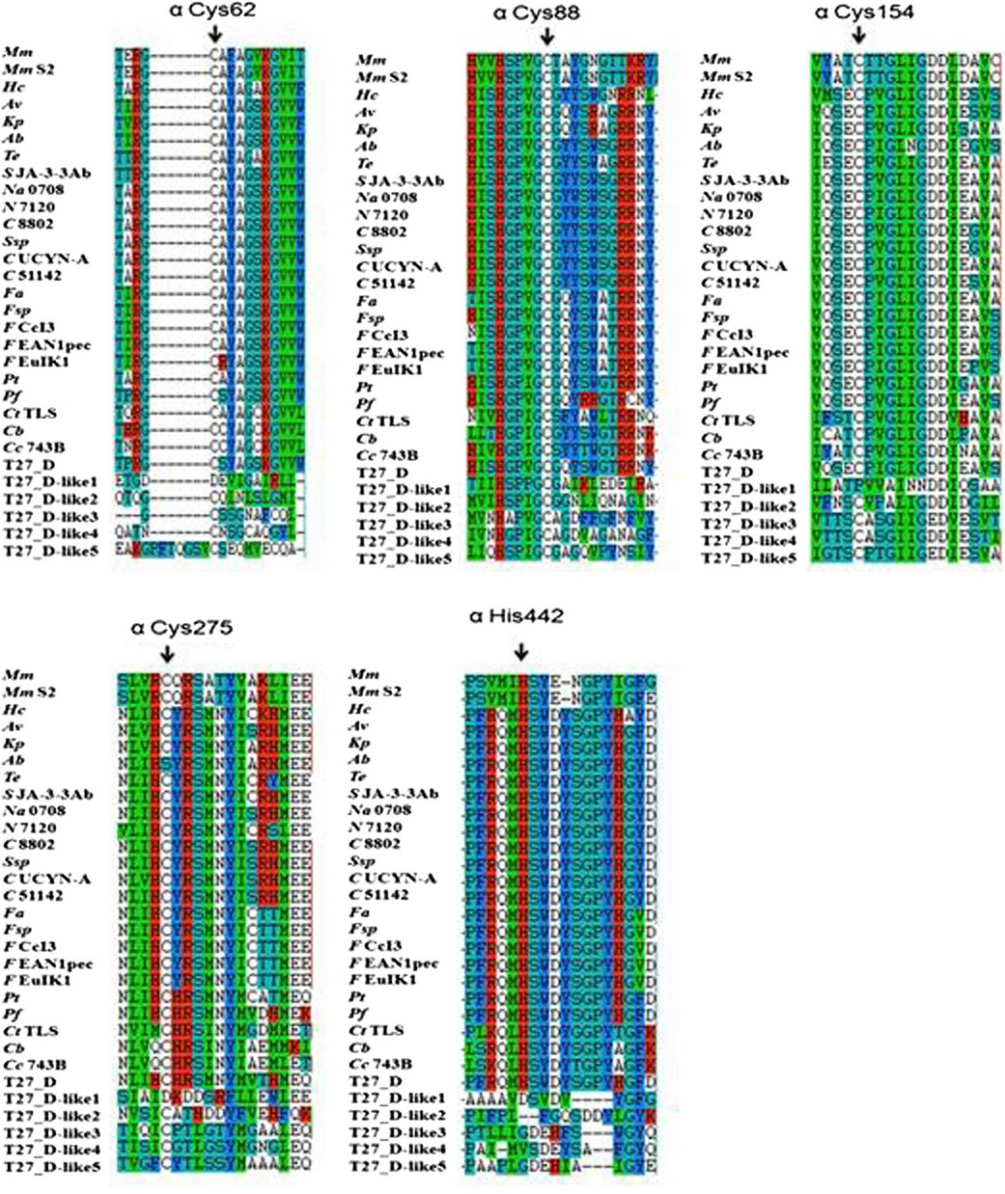
**Conservation in and around crucial residues (FeMo-co and P-cluster ligands) in NifD and NifD-like protein sequences from** ***P. sabinae*** **The P-cluster and FeMo-co ligands, based on** ***A vinelandii*** **numbering, are indicated with vertical arrows.** *Ab* = *A. brasilense*, *Av* = *A. vinelandii*, *Ct* TLS = *C. tepidum* TLS, *Cb* = *Clostridium beijerinckii*, *Cc* 743B = *C. cellulovorans* 743B, *C* UCYN-A = *Cyanobacterium* UCYN-A, *C* 51142 = *C*. sp. ATCC 51142, *C* 8802 = *C*. sp. PCC 8802, *Fal* = *F. alni* ACN14a, *Fsp* = *Frankia* sp., *F* CcI3 = *F*. sp. CcI3, *F* EAN1pec = *F*. sp. EAN1pec, *F* EuIK1 = *F*. sp. EuIK1, *Hc* = *H.chlorum*, *Kp* = *K. pneumoniae* 342, *Mm* = *M. maripaludis*, *Mm* S2 = *M. maripaludis* S2, *Na* 0708 = *N. azollae* 0708, *N* 7120 = *N*. sp. PCC 7120, *Pf* = *P. fujiensis*, *Pt* = *P. terrae* HPL-003, *Ssp* = *S*. sp., *S* JA-3-3Ab = *S*. sp. JA-3-3Ab, *Te* = *T. erythraeum* IMS101.

**Figure 8:**
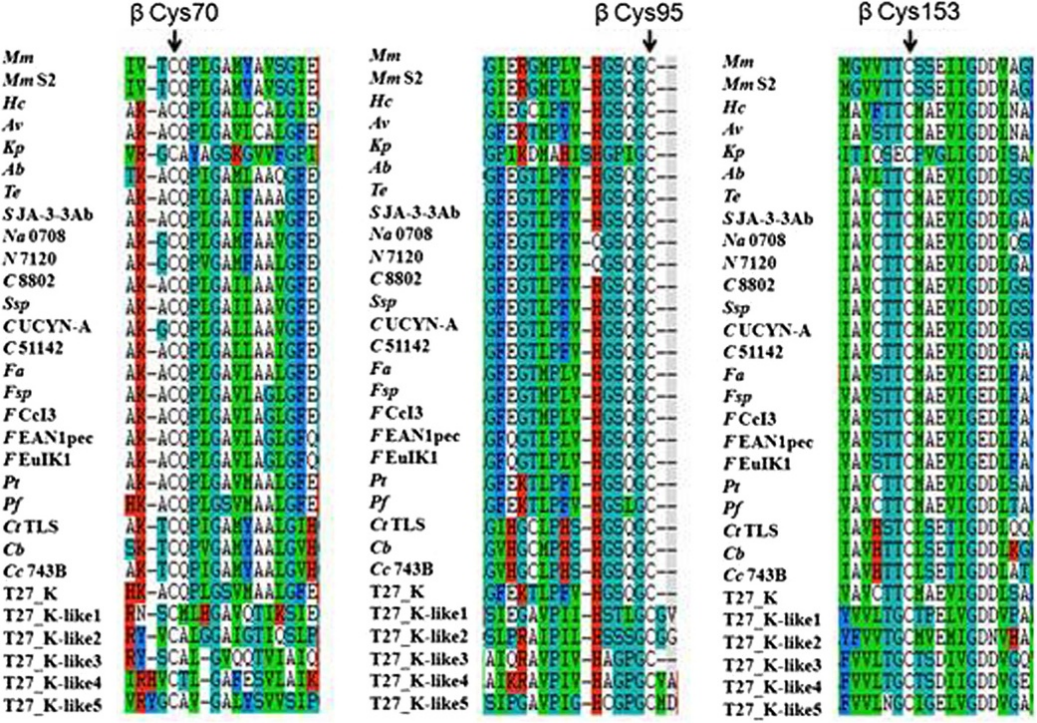
**Conservation in and around crucial residues (P-cluster ligands) in NifK and NifK-like protein sequences from** ***P. sabinae*** **T27.** The P-cluster ligands based on *A. vinelandii* numbering, are indicated with vertical arrows. *Ab* = *A. brasilense*, *Av* = *A. vinelandii*, *Ct* TLS = *C. tepidum* TLS, *Cb* = *Clostridium beijerinckii*, *Cc* 743B = *C. cellulovorans* 743B, *C* UCYN-A = *Cyanobacterium* UCYN-A, *C* 51142 = *C*. sp. ATCC 51142, *C* 8802 = *C. sp*. PCC 8802, *Fal* = *F. alni* ACN14a, *Fsp* = *Frankia* sp., *F* CcI3 = *F*. sp. CcI3, *F* EAN1pec = *F*. sp. EAN1pec, *F* EuIK1 = *F*. sp. EuIK1, *Hc* = *H.chlorum*, *Kp* = *K. pneumoniae* 342, *Mm* = *M. maripaludis*, *Mm* S2 = *M. maripaludis* S2, *Na* 0708 = *N. azollae* 0708, *N* 7120 = *N*. sp. PCC 7120, *Pf* = *P. fujiensis*, *Pt* = *P. terrae* HPL-003, *Ssp* = *S*. sp., *S* JA-3-3Ab = *S*. sp. JA-3-3Ab, *Te* = *T. erythraeum* IMS101.

The *nifHDK* are structural genes of Mo-nitrogenase, with the *nifD* and *nifK* genes encoding the α and β subunits, respectively, of the molybdenum iron protein (dinitrogenase) and the *nifH* the γ subunit of the iron protein (dinitrogenase reductase). The genome of *P. sabinae* T27 contains two *nifH*-like, five *nifD*-like and five *nifK*-like genes. Conserved residues in alignments of NifH-like sequences (Figure 6) with NifH sequences show that 4Fe-4S iron sulfur cluster-ligating cysteines and the P-loop/MgATP binding motif are invariant, suggesting that these proteins may function analogously to dinitrogenase reductase. Conversely, NifD/NifK-like sequences are highly diverged from both the nitrogenase subunits. FeMoco-ligating residues at αCys275 and αHis442 of NifD (Figures 7, 8) are not conserved in NifD/NifK-like sequences, although several—but not all—conserved cysteines involved with P-cluster coordination are found in NifD/Nifk-like sequences.

#### Expressions of *nifHDK*and *nifHDK*-like genes in N_2_-fixing and non-N_2_-fixing conditions

**Figure 9:**
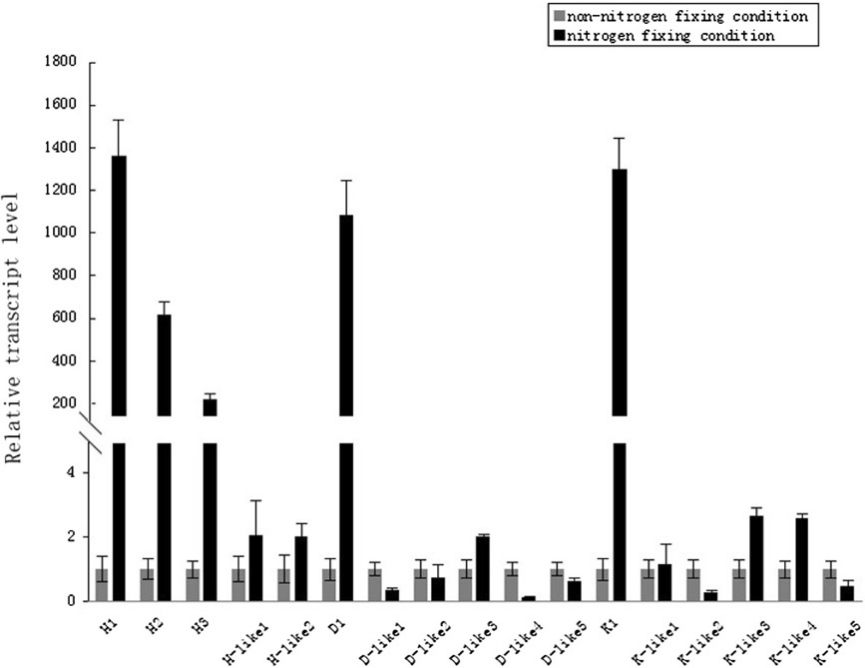
**Quantitative real-time RT-PCR analysis of transcripts of** ***nifHDK*** **and** ***nifHDK-*** **like genes of** ***P. sabinae*** **T27.**

It is generally recognized that *nif* genes are expressed in N_2_-fixing conditions (the microaerobic or anaerobic and without ammonium or limited ammonium). In order to examine whether the transcription of *nifHDK* and *nifHDK*-like genes is regulated by ammonium and oxygen in *Paenibacillus*, expression levels of the *P. sabinae* T27 *nifH*, *nifD*, *nifK*, *nifH*-*like*, *nifD*-*like* and *nifK*-*like* genes were detected by the real-time quantitative RT-PCR method using RNA isolated from cells grown under N_2_-fxing and non-N_2_-fxing conditions. As shown in Figure 9, a large (200–1300 fold) increase in the transcript levels of the *nifH*, *nifD* and *nifK* genes was observed in N_2_-fixing conditions compared to those in the non- N_2_ fixing conditions. Especially, *nifH1*, *nifD1* and *nifK1* within the complete *nif* cluster were significantly expressed in N_2_-fixing conditions compared to those in the non- N_2_ fixing conditions. The data are consistent with the previous reports that the transcription of *nifHDK* genes is regulated by ammonium and oxygen in N_2_-fixing organisms, suggesting that the *nifHDK* genes of *P. sabinae* T27 are involved in nitrogen fixation. In contrast to *nifHDK*, *nifHDK*-*like* genes of *P. sabinae* T27 were not significantly differently expressed in N_2_-fixing and non-N_2_-fixing conditions, suggesting that these *nif*-like genes did not function in nitrogen fixation.

#### Functional analysis of nifH/nifH-like and nifD/nifD-like genes in nitrogen fixation

**Figure 10:**
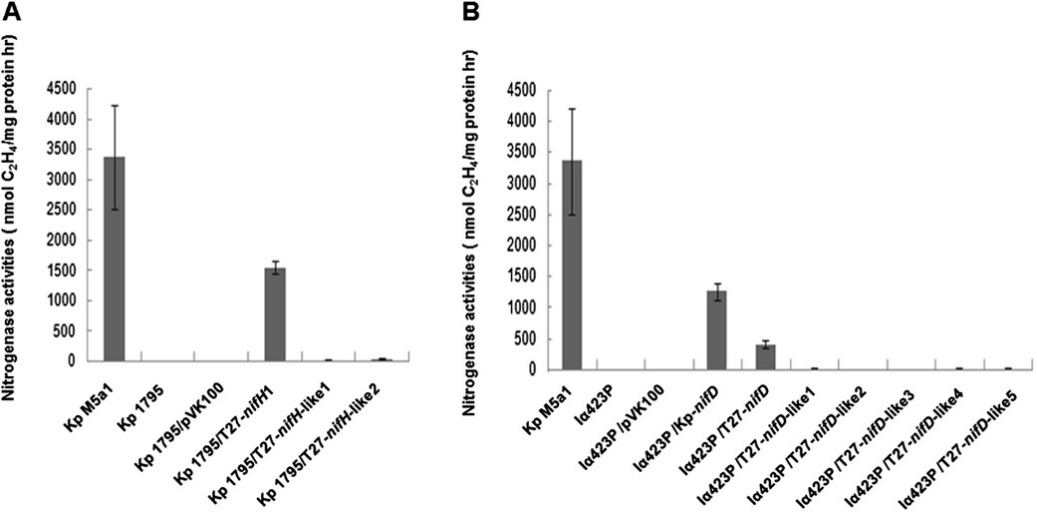
**The nitrogenase activities of complementary strains and control strains.** Kp M5a1: Wild-type *K. pneumonia*; Kp1795: *K. pneumonia nifH* mutant strain; Iα423P: *K. pneumonia nifD* mutant strain. **(A)** Complementation by *nifH*/*nifH*-like genes. **(B)** Complementation by *nifD*/*nifD*-like genes.

To further comparatively study the functions of the nif and nif-like gene of P. sabinae T27, K. *pneumonia nifH*^−^ mutant strain 1795 and *nifD* mutant strain Iα423P [26], both of which have no or very low nitrogenase activity, were complemented with the *nifH*/*nifH*-*like* and *nifD*/*nifD*-like genes of *P. sabinae* T27 under the control of *K. pneumonia nifH* promoter, respectively. As shown in Figure 10A, the complementary strains carrying *nifH*-like1 or *nifH*-like2 of *P. sabinae* T27 could not resumed the nitrogenase activity of *K. pneumonia nifH*^−^ mutant strain 1795, while the *nifH1* from the complete *nif* cluster of *P. sabinae* T27 could restore to nearly 50% of the wild-type strain M5al. The data are consistent with our previous report that the three copies of *nifH* could restore nitrogenase activity of *K. pneumonia nifH*^−^ mutant strain 1795 [27]. Likewise, *nifD* of *P. sabinae* T27 could resumed the nitrogenase activity of *K. pneumonia nifD*^−^ mutant strain Iα423P, although *K. pneumonia nifD* enabled *K. pneumonia nifD*^−^ mutant strain Iα423P to have higher nitrogenase activity than *P. sabinae* T27 *nifD* did (Figure 10B). In contrast, none of *nifD*-like1, *nifD*-like2, *nifD*-like3, *nifD*-like4 and *nifD*-like5 could restore the nitrogenase activity of *K. pneumonia nifD*^−^ mutant. These data suggest that *nif*-like genes may be not involved in nitrogen fixation.

### The complete *nif*gene cluster is organized as an operon

Bioinformatics analysis revealed that the ten genes *nifBHDKENXorf1hesAnifV* within the complete *nif* gene cluster are organized as an operon. Here RT-PCR experiments using primers designed to span across intergenic regions indicated that the nine genes within the *nif* cluster are organized in a single operon (Additional file 7: Figure S7). Single operon *nif* clusters have been reported in gram-positive prokaryotes and in the archaea, e.g. *Heliobacterium chlorum*[28] and *Methanococcus maripaludis*[29]. However, in contrast to these *nif* clusters *P. sabinae* T27 does not contain the negative regulatory genes *nifI1* and *nifI2* (homologues of *glnB*), which are involved in post-translational regulation of nitrogenase activity in response to fixed nitrogen [30].

### The complete *nif*gene cluster of *P. sabinae*T27 has a σ^70^-dependent promoter

Almost all of the *nif* genes in Gram-negative nitrogen-fixing bacteria, such as *K. pneumoniae* and *A. vinelandii*, are transcribed from σ^54^ promoters (−24/-12) whose expression depends on activator NifA [31]. However, the presumed promoter regions for the *nif* genes of *P. sabinae* T27 have sequences which are similar to the *E. coli* σ^70^-dependent −35 and −10 consensus promoter. The following experiments demonstrated that the *nif* promoter of *P. sabinae* T27 is distinct from those of those of Gram-negative nitrogen-fixing bacteria.

The transcriptional start site (TSS) of the *nif* gene cluster in *P. sabinae* T27 was determined by using the 5′-RACE (Rapid Amplification of cDNA Ends) method. The TSS was located 222 bp upstream of the translational start site of *nifB* and a putative promoter was identified 6 nucleotides preceding the TSS (Additional file 8: Figure S8A). The −35 (TTGACG) and −10 (TATGAT) sequences in the *nifB* promoter were similar to the corresponding consensus sequences (TTGACA and TATAAT respectively) of *E. coli* σ^70^-dependent promoters. A σ^54^-dependent −24/-12 promoter sequence was not observed upstream of the *nif* cluster. Downstream of *nifV*, a potential transcriptional termination site was identified, containing two potential stem loops followed by a T-rich region (Additional file 8: Figure S8A). These findings indicate that the *nif* genes in *P. sabinae* T27 are organized as a single operon containing 9 genes, which is transcribed from an *rpoD*-dependent promoter.

To analyze the σ^70^-dependentcy of the *nifB* promoter, electrophoretic mobility shift assays (EMSA) were carried out using either *E. coli* σ^70^-RNAP (RNA polymerase) or σ^70^ from *P. sabinae* T27, which was overexpressed and purified from *E. coli* (Additional file 8: Figure S8B). EMSA experiments revealed that both purified σ^70^ from *P. sabinae* T27 and *E. coli* σ^70^-RNAP holoenzyme bind to the 45 bp *nifB* promoter fragment. Competition experiments with non-labelled *nifB* DNA indicated that the *E. coli* RNAP holoenzyme binds more tightly to this DNA fragment, since higher concentrations of competitor were apparently required to dissociate the *E. coli* σ^70^-RNAP (Additional file 8: Figure S8C and D). These results are consistent with the ability of σ^A^ (σ^70^) of *Bacillus subtilis* to bind to promoters independent of core RNAP [32, 33].

### The complete *nif*gene cluster of *P. sabinae*T27 enables *E. coli*to fix nitrogen

We further cloned the 12-kb full-length *nif* gene cluster consisting of its own *nif* promoter and the contiguous nine genes *nifBHDKENXorf1hesAnifV* into the wide-host plasmid pVK100 and then transformed this into *E. coli* JM109, yielding the recombinant *E. coli* strain 27 (Additional file 9: Figure S9). To determine whether the *Paenibacillus nif* gene cluster functions in *E. coli*, we employed two independent methods to assess nitrogenase activity: firstly, reduction of the alternative substrate acetylene to ethylene, which can be readily quantified by gas chromatography [34, 35] and secondly, a ^15^ N_2_ enrichment assay to directly measure the incorporation of this tracer into organic nitrogen [36]. When grown anaerobically in nitrogen-deficient medium, *P. sabinae* T27 exhibits both acetylene reduction and ^15^ N_2_ incorporation (Additional file 9: Figure S9). The recombinant *E. coli* strain 27, which expresses the *nif* genes from the native promoter showed approximately 10% of the specific activity for acetylene reduction when compared with *Paenibacillus* and was competent to assimilate ^15^ N_2_. The results demonstrated that the complete *nif* gene cluster is a functional unit.

## Conclusions

In this study, we uncovered the contents and organization of nif and nif-like genes of *P. sabinae* T27 by completing its genome sequence. The genome of *P. sabinae* T27 contains fifteen nitrogen fixation (*nif*) genes, including three *nifH*, one *nifD*, one *nifK*, four *nifB*, two *nifE*, two *nifN*, one *nifX* and one *nifV*. Of the 15 *nif* genes, eight *nif* genes (*nifB*, *nifH*, *nifD*, *nifK*, *nifE*, *nifN*, *nifX* and *nifV*) and two non-*nif* genes (*orf1* and *hesA*) form a complete *nif* gene cluster. Phylogenetic analysis suggests that the complete *nif* cluster of *P. sabinae* T27 was originated from a common ancestor with *Frankia*. Multiple *nifB*, *nifH*, *nifE*, *nifN* may result from duplication. The complete *nif* gene cluster is organized in an operon as a functional unit for nitrogen fixation. The complete *nif* gene cluster under the control of its σ^70^-dependent promoter enabled *Escherichia coli* JM109 to fix nitrogen. *P. sabinae* T27 contains two *nifH*-like genes and five pairs of *nifDK*-like genes. Unlike *nif* genes, the transcriptions of *nifHDK*-like genes were not regulated by ammonium and oxygen and *nifHDK*-like genes were not involved in nitrogen fixation.

## Methods

### Strains and media

Strains used in this study is listed in Additional file 10: Table S1. *P. sabinae* T27 and the recombinant *E. coli* strains were routinely grown in LD medium (per liter contains: 2.5 g NaCl, 5 g yeast and 10 g tryptone) at 30°C with shaking. When appropriate, antibiotics were added in the following concentrations: 100 μg∕ml ampicillin, and 20 μg∕ml tetracycline for maintenance of plasmids.

Nitrogen-free and nitrogen-deficient media were used in this study. Nitrogen-free medium contained (per liter) 10.4 g Na_2_HPO_4_, 3.4 g KH_2_PO_4_, 26 mg CaCl_2_• 2H_2_O, 30 mg MgSO_4_, 0.3 mg MnSO_4_, 36 mg Ferric citrate, 7.6 mg Na_2_MoO_4_ · 2H_2_O, 10 μg p-aminobenzoic acid, 5 μg biotin and 4 g glucose as carbon source. Nitrogen-deficient medium contained 2 mM glutamate as nitrogen source in nitrogen-free medium [20].

### Phylogenetic analysis

Maximum-likelihood (ML) phylogenetic trees were constructed using PhyML (version 3.0) [37] software and multiple alignment of amino acid sequences were carried out by ClustalW (version 2.1) [38].

### Genome sequencing, genome annotation and analysis

Genomic DNA of *P. sabinae* T27 was isolated according to [13]. Genome sequencing was performed by Tianjin Research Center for Functional Genomics and Biochip in China. The genome *P. sabinae* T27 was sequenced by using a hybrid sequencing approach that incorporates 454 pyrosequencing with Illumina Genome Analyzer. Sequencing by both methods was performed according to manufacturer’s instructions, Roche and Illumina.

The rRNA genes were identified with RNAmmer [39]. Transfer RNA (tRNA) genes were identified by the program tRNAscan-SE [40]. Genes coding for proteins with known functions were annotated by searches against KEGG Genes, Pfam, and SWISSPROT [41]. The complete sequence has been assigned GenBank accession no. CP004078.

### Construction of recombinant plasmid for expression of the complete *nif*cluster in *E. coli*

Genomic DNA of *P. sabinae* T27 was used as template for cloning *nif* genes. A 12 kb Xho I-Xho I DNA fragment containing the complete *nif* gene cluster (a 310 bp promoter region and the contiguous ten genes *nifBHDKENXorf1hesAnifV* and 194 bp downstream of the stop codon TAA of *nifV*) was PCR amplified with primers T-up and T-down (Additional file 11: Table S2). The PCR product was ligated to Xho I site of pVK100, yielding plasmid pKY100-27. Then the plasmid was transferred to *E. coli* JM109, yielding the recombinant *E. coli* 27 strain.

### Construction of plasmids for complementation studies

In order to determine the function of *nifH*/*nifH*-like and *nifD*/*nifD*-like genes, overlap PCR was performed to fuse the coding regions of *nifH1*, *nifH*-like1, *nifH*-like2, *nifD*, *nifD*-like1, *nifD*-like2, *nifD*-like3, *nifD*-like4 and *nifD*-like5 of *P. sabinae* T27 with the *nifH* promoter of *K. pneumoniae*. The primers used in fusion were listed in Additional file 11: Table S2. The amplified PCR products were cloned to pVK100. The recombinant pVK100 were transformed to *K. pneumoniae nifH* mutant or *K. pneumoniae nifD* mutant for complementation.

### Transcription start site identification

The 5′-RACE method was used to determine the transcription start site (TSS) using the SMARTer™ RACE cDNA Amplification Kit (Clontech). Gene-specific primers are listed in Additional file 11: Table S2. The PCR product was cloned into the pMD18-T Vector and then sequenced.

### Overexpression and purification of σ^70^from *P. sabinae*T27 in *E. coli*

A 1134 bp DNA fragment carrying the *rpoD* gene (encoding σ^70^ of *P. sabinae* T27) was PCR amplified with primers sigma A-F and sigma A-R (Additional file 11:Table S2). The PCR product was ligated to the pET-28b expression vector, yielding plasmid pET28-σ^70^. *E. coli* strain BL21 (DE3) was transformed with expression plasmid pET28-σ^70^ and utilized for protein expression. The bacterial cells were grown in LB medium to the end of log phase and then a final concentration of 1 mM IPTG (isopropyl-β-D-thiogalactopyranoside) was added to the culture and the cells were harvested after incubation for another 4 h at 16°C. The cells were then harvested and disrupted by sonication on ice. The protein was purified from the supernatant with Ni^2+^-NTA agarose (Qiagen) according to the manufacturer’s instructions.

### Electrophoretic mobility shift assay (EMSA)

For the electrophoretic mobility shift assay (EMSA), a 50 bp *nif* promoter fragment (from −47 to +3 relative to the transcription start site of *nifB* in *P. sabinae* T27) was synthesized by Sangon Biotech Co., Ltd (Shanghai). To do this, two DNA fragments corresponding to the sequences of the first strand (5′- GGAGAAGTGAATTGACTGTATTTGTCCCTGTCTCTAAGA-TGTAATTATAT-3′) and the complementary DNA strand (5′- ATATAATTACATCTTAGAGAC-AGGGACAAATACAGTCAATTCACTTCTCC-3′) were synthesized. The two strands were annealed and then labeled with digoxin using the DIG Gel Shift Kit (Roche). The binding shift experiment of *E. coli* σ^70^-RNAP (RNA polymerase) (Epicentre) or σ^70^ of *P. sabinae* T27 to the *nif* promoter was carried out using a gel shift kit (Roche). At the same time, a scrambled 39 bp DNA fragment formed by annealing the following complementary oligonucleotides (5′- GTACGGAGTATCCAGCTCCGTAGCATGCAAATCCTCTGG-3′) and (5′-CCAGAGGATTTGCATGCTACGGAGCTGGATACTCCGTAC -3′) was used to assay non-specific binding.

### RT-PCR and qRT-PCR analysis

For RT-PCR, *P. sabinae* T27 was grown in N_2_-fixing conditions (without NH_4_Cl and O_2_). For qRT-PCR, *P. sabinae* T27 was grown in N_2_-fixing conditions (without NH_4_Cl and O_2_) and non- N_2_-fixing conditions (100 mM ammonium and 21% O_2_). The culture was harvested by centrifugation at 4 °C, and total RNA was isolated using the PrimeScript® RT reagent Kit with gDNA Eraser (Takara Bio) according to the manufacturer’s instructions. The possibility of contamination of genomic DNA was eliminated by digestion with RNase-free DNase I (Takara Bio). The integrity and size distribution of the RNA was verified by agarose gel electrophoresis, and the concentration was determined spectrophotometrically. Synthesis of cDNA was carried out using RT Prime Mix according to the manufacturer’s specifications (Takara Bio). 0.8 μg of cDNA was used for RT-PCR. The *nif* and *nif*-like gene transcripts were detected by using an RT-PCR Kit with 16S rDNA as a control. Primers for *nif*, *nif*-like genes and 16S rDNA used for PCR are listed in (Additional file 11: Table S2).

### Nitrogenase activity assays by acetylene reduction method

For nitrogenase activity assays, *P. sabinae* T27 and the recombinant *E. coli* 27 strain were grown in 5 mL of LD media (supplemented with antibiotics when necessary) in 50-ml flasks shaken at 250 rpm for 16 h at 30°C. Nitrogenase activity assays was performed according to Wang et al’s reports [20].

### ^15^ N_2_ incorporation assay

*P. sabinae* T27 and the recombinant *E. coli* strain were grown overnight in LD medium. The cultures were collected and resuspended in 70 ml nitrogen-deficient medium containing 2 mM glutamate as nitrogen source to an OD_600_ of 0.4 in a 120 ml serum bottle. ^15^ N_2_ incorporation assay was performed according to Wang et al’s report [20].

## Electronic supplementary material

Additional file 1: Figure S1: Schematic overview of metabolic pathways and transport systems in *P. sabinae* T27. Predicted transporters are grouped by energy specificity: red, ATP-dependent transporters; deep pink, symporters; light pink, ion channels; yellow, transporter family. Arrows indicate direction of transport. Final biosynthetic products are indicated with orange boxes. Crosses indicate pathways or reactions that are apparently not present in *P. sabinae* T27. (JPEG 500 KB)

Additional file 2: Figure S5: Maximum-likelihood tree based on complete HesA protein sequences showing relationships between HesA protein of *P. sabinae* T27 and HesA proteins from representative microorganisms. The numbers at the nodes indicate levels of bootstrap support (%) based on a neighbor-joining analysis of 100 resampled datasets; only values at or above 50% are given, Bar 0.1 substitutions per amino acid position. (JPEG 172 KB)

Additional file 3: Figure S6: Maximum-likelihood tree based on ORF1 sequences showing relationships between ORF1 of *P. sabinae* T27 and ORF1 from representative microorganisms. The numbers at the nodes indicate levels of bootstrap support (%) based on a neighbor-joining analysis of 100 resampled datasets; only values at or above 50% are given, Bar 0.1 substitutions per amino acid position. (JPEG 129 KB)

Additional file 4: Figure S2: Maximum-likelihood tree based on complete NifB protein sequences showing relationships between NifB proteins of *P. sabinae* T27 and NifB proteins from representative microorganisms. The numbers at the nodes indicate levels of bootstrap support (%) based on a neighbor-joining analysis of 100 resampled datasets; only values at or above 50% are given, Bar 0.1 substitutions per amino acid position. (JPEG 247 KB)

Additional file 5: Figure S3: Maximum-likelihood tree based on complete NifH protein sequences showing relationships between NifH-like proteins of *P. sabinae* T27 and NifH proteins from representative microorganisms. The numbers at the nodes indicate levels of bootstrap support (%) based on a maximum-likelihood analysis of 100 resampled datasets; only values at or above 50% are given, Bar 0.2 substitutions per amino acid position. (JPEG 214 KB)

Additional file 6: Figure S4: Maximum-likelihood tree based on complete NifEN protein sequences showing relationships between NifEN proteins of *P. sabinae* T27 and NifEN proteins from representative microorganisms. The numbers at the nodes indicate levels of bootstrap support (%) based on a neighbor-joining analysis of 100 resampled datasets; only values at or above 50% are given, Bar 0.1 substitutions per amino acid position. *Paenibacillus sabinae* T27 NifE1N1 (AHV98644, AHV98643), *Paenibacillus sabinae* T27 NifE2N2 (AHV98966, AHV98965), *Cyanobacterium* UCYN-A (YP_003421699, YP_003421700), *Cyanothece sp*. ATCC 51142 (ACB49914, ACB49915), *Frankia sp*. CcI3 (YP_483560, YP_483559), *Nostoc azollae* 0708 (YP_003720729, YP_003720728), *Nostoc punctiforme* PCC 73102 (YP_001869140, YP_001869141), *Nostoc sp*. PCC 7120 (WP_010995610, WP_010995609), *Synechococcus sp*. JA-3-3Ab (YP_475248, YP_475249), *Azospirillum brasilense* (WP_014199505, WP_014199506), *Azotobacter vinelandii* (AAA64716, AAA64717), *Chlorobium tepidum* TLS (NP_662422, NP_662423), *Trichode*s*ium erythraeum* IMS101 (YP_723620, YP_723620), *Cyanothece sp*. PCC 8802 (YP_003137550, YP_003137551), *Frankia sp*. EuIK1 (AAD17262, AAD17263), *Frankia sp*. EAN1pec (ABW16212, ABW16211), *Frankia alni* ACN14a (YP_716936, YP_716935), *Heliobacterium chlorum* (BAD95756, BAD95757), *Klebsiella pneumoniae* 342 (YP_002237560, YP_002237559). *Methanococcus maripaludis* (AAC45517, AAC45518), *Paenibacillus terrae* HPL-003 (YP_005075595, YP_005075596), *Methanococcus maripaludis* S2 (NP_987978, NP_987979), *Methanococcus vannielii* SB (YP_001322586, YP_001322585), *Methanothermobacter thermautotrophicus* str. Delta H (NP_276678, NP_276679), *Paenibacillus sp*. Aloe-11 (WP_007429045, WP_007429046), *Paenibacillus massiliensis* (AAX73208, AAX73209), *Rivularia sp*. PCC 7116 (YP_007059114, YP_007059113). (JPEG 101 KB)

Additional file 7: Figure S7: The ten genes *nifB1*, *nifH1*, *nifD*, *nifK*, *nifE1*, *nifN1*, *nifX*, *orf1*, *hesA* and *nifV* within the complete *nif* gene cluster of *P. sabinae* T27 are organized in an operon as determined by RT-PCR. **(A)** Outline of the strategy. Primers used and amplified products (numbered) are given below the schematic representation of the genes. **(B)** Result of RT-PCR reactions with RNA from *P. sabinae* T27 grown under N_2_-fixing conditions. The numbering on the top of the gels corresponds to the product numbers drawn schematically in the outline given above. RT, standard RT-PCR reaction; (−), negative control in which no reverse transcriptase was added to the RT reaction; (+), positive control in which genomic DNA was used as template in the RT-PCR. (JPEG 80 KB)

Additional file 8: Figure S8: Characterization of the *nif* promoter of *P. sabinae* T27. (A) Nucleotide sequence of the *nifB* promoter. (B) Overexpression and purification of σ^70^ from Lane 1: protein marker; lane 2: uninduced protein; lane 3: induced protein; lanes 4: purified σ^70^ factor. (C) Electrophoretic mobility shift assays (EMSA) demonstrating binding of *P. sabinae* σ^70^ to the 45 bp *nifB* promoter DNA fragment (final concentration 0.03 pmol). The protein concentration is indicated in pmol above each lane (left hand panel). In the right hand panel, the protein concentration was maintained at 2.4 pmol and unlabeled *nifB* promoter fragment was added as competitor (concentration indicated above each lane). (D) EMSA experiments demonstrating binding of *E. coli* σ^70^-RNAP to the 45 bp *nifB* promoter DNA fragment (final concentration 0.03). The protein concentration is indicated in pmol above each lane (left hand panel). In the right hand panel, the protein concentration was maintained at 0.2 pmol and unlabeled *nifB* promoter fragment was added as competitor (concentration indicated above each lane). (JPEG 111 KB)

Additional file 9: Figure S9: Nitrogenase activity of *E. coli* T27, *E. coli* JM109 and *P. sabinae* T27. *E. coli* T27 carrying the complete *nif* gene cluster from *P. sabinae* T27. *E. coli* JM109 carrying the empty vector plasmid pVK100 and *P. sabinae* T27 are used as negative and positive controls, respectively. Strains were grown anaerobically in nitrogen-deficient conditions. (TIFF 647 KB)

Additional file 10: Table S1: Strains and plasmids used in this research. (DOCX 17 KB)

Additional file 11: Table S2: Primers used in this study. (DOCX 24 KB)
