# Supplementary figures and images for: The genome of Paenibacillus sabinae T27 provides insight into evolution, organization and functional elucidation of nif and nif-like genes
Source: BMC Genomics. 2014 Aug 27;15(1):723. doi: 10.1186/1471-2164-15-723 (PMC4246453; doi:10.1186/1471-2164-15-723)

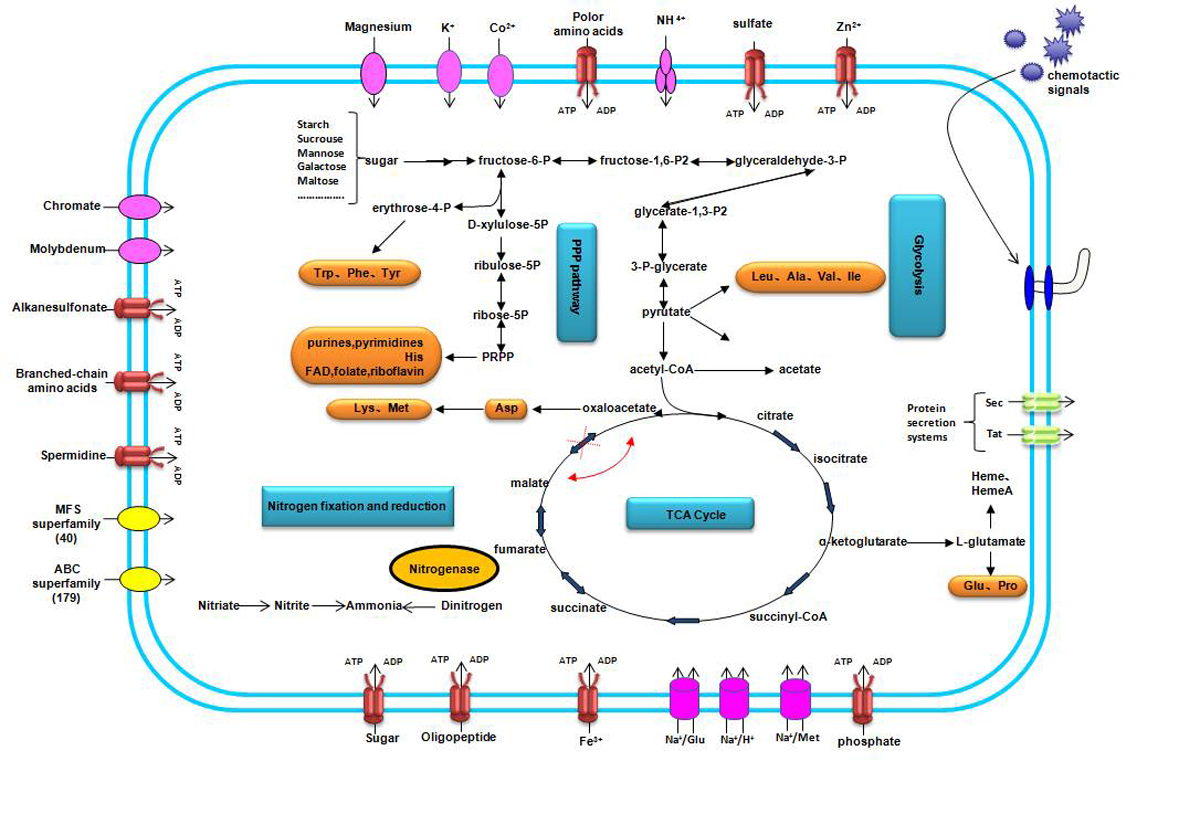

Supplement: Supplementary file 1 — Additional file 1: Figure S1: Schematic overview of metabolic pathways and transport systems in P. sabinae T27. Predicted transporters are grouped by energy specificity: red, ATP-dependent transporters; deep pink, symporters; light pink, ion channels; yellow, transporter family. Arrows indicate direction of transport. Final biosynthetic products are indicated with orange boxes. Crosses indicate pathways or reactions that are apparently not present in P. sabinae T27. (JPEG 500 KB) [file 12864_2013_6682_MOESM1_ESM.jpeg]

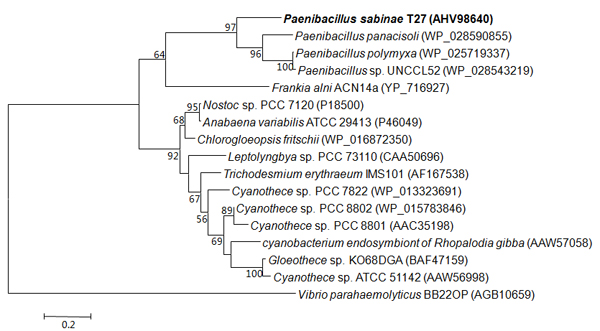

Supplement: Supplementary file 2 — Additional file 2: Figure S5: Maximum-likelihood tree based on complete HesA protein sequences showing relationships between HesA protein of P. sabinae T27 and HesA proteins from representative microorganisms. The numbers at the nodes indicate levels of bootstrap support (%) based on a neighbor-joining analysis of 100 resampled datasets; only values at or above 50% are given, Bar 0.1 substitutions per amino acid position. (JPEG 172 KB) [file 12864_2013_6682_MOESM2_ESM.jpeg]

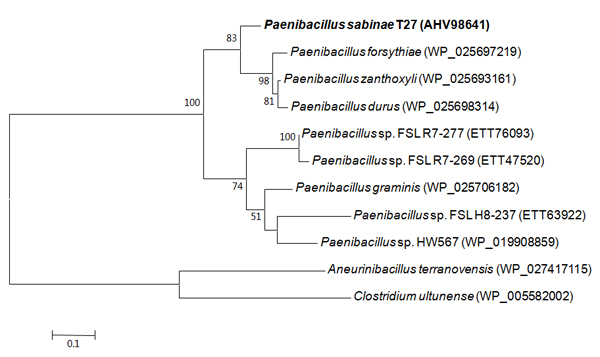

Supplement: Supplementary file 3 — Additional file 3: Figure S6: Maximum-likelihood tree based on ORF1 sequences showing relationships between ORF1 of P. sabinae T27 and ORF1 from representative microorganisms. The numbers at the nodes indicate levels of bootstrap support (%) based on a neighbor-joining analysis of 100 resampled datasets; only values at or above 50% are given, Bar 0.1 substitutions per amino acid position. (JPEG 129 KB) [file 12864_2013_6682_MOESM3_ESM.jpeg]

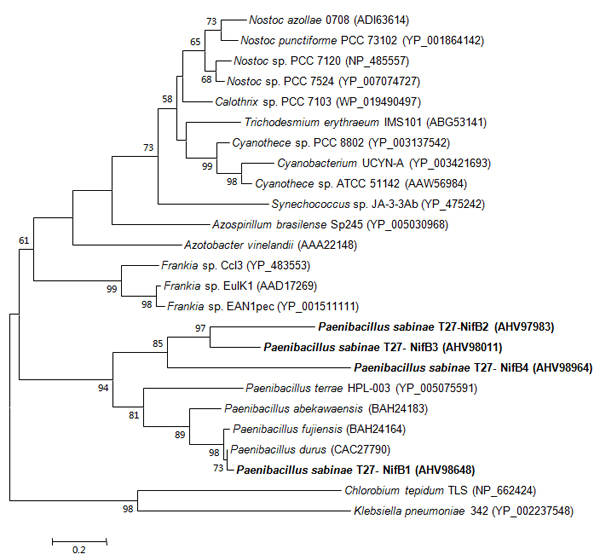

Supplement: Supplementary file 4 — Additional file 4: Figure S2: Maximum-likelihood tree based on complete NifB protein sequences showing relationships between NifB proteins of P. sabinae T27 and NifB proteins from representative microorganisms. The numbers at the nodes indicate levels of bootstrap support (%) based on a neighbor-joining analysis of 100 resampled datasets; only values at or above 50% are given, Bar 0.1 substitutions per amino acid position. (JPEG 247 KB) [file 12864_2013_6682_MOESM4_ESM.jpeg]

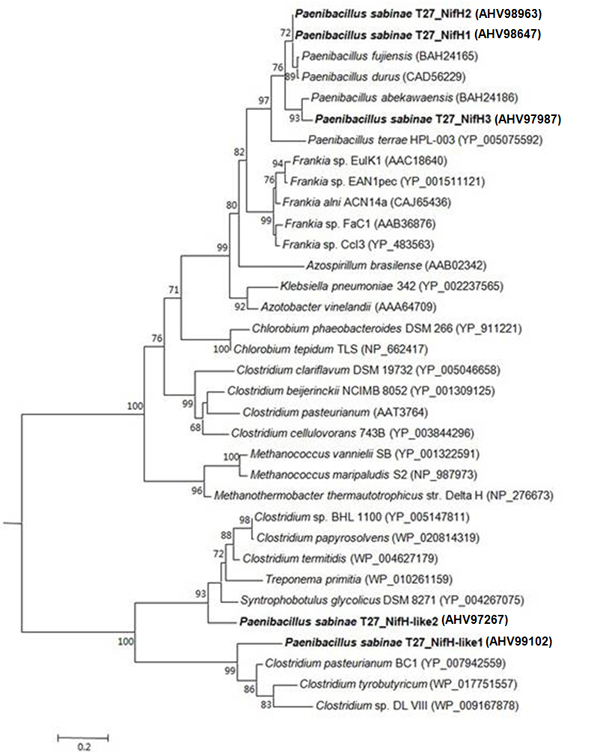

Supplement: Supplementary file 5 — Additional file 5: Figure S3: Maximum-likelihood tree based on complete NifH protein sequences showing relationships between NifH-like proteins of P. sabinae T27 and NifH proteins from representative microorganisms. The numbers at the nodes indicate levels of bootstrap support (%) based on a maximum-likelihood analysis of 100 resampled datasets; only values at or above 50% are given, Bar 0.2 substitutions per amino acid position. (JPEG 214 KB) [file 12864_2013_6682_MOESM5_ESM.jpeg]

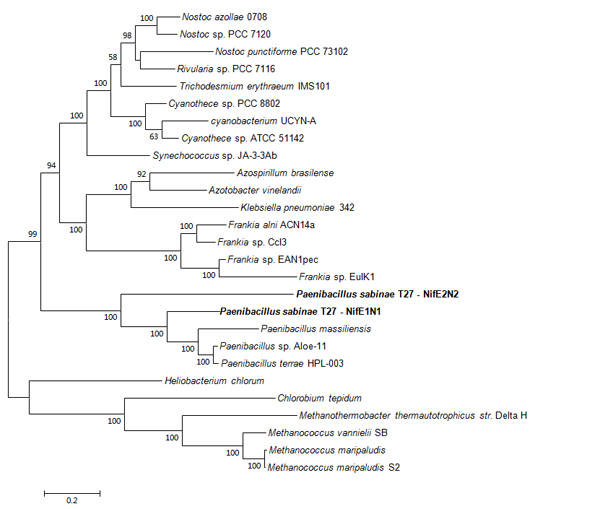

Supplement: Supplementary file 6 — Additional file 6: Figure S4: Maximum-likelihood tree based on complete NifEN protein sequences showing relationships between NifEN proteins of P. sabinae T27 and NifEN proteins from representative microorganisms. The numbers at the nodes indicate levels of bootstrap support (%) based on a neighbor-joining analysis of 100 resampled datasets; only values at or above 50% are given, Bar 0.1 substitutions per amino acid position. Paenibacillus sabinae T27 NifE1N1 (AHV98644, AHV98643), Paenibacillus sabinae T27 NifE2N2 (AHV98966, AHV98965), Cyanobacterium UCYN-A (YP_003421699, YP_003421700), Cyanothece sp. ATCC 51142 (ACB49914, ACB49915), Frankia sp. CcI3 (YP_483560, YP_483559), Nostoc azollae 0708 (YP_003720729, YP_003720728), Nostoc punctiforme PCC 73102 (YP_001869140, YP_001869141), Nostoc sp. PCC 7120 (WP_010995610, WP_010995609), Synechococcus sp. JA-3-3Ab (YP_475248, YP_475249), Azospirillum brasilense (WP_014199505, WP_014199506), Azotobacter vinelandii (AAA64716, AAA64717), Chlorobium tepidum TLS (NP_662422, NP_662423), Trichodesium erythraeum IMS101 (YP_723620, YP_723620), Cyanothece sp. PCC 8802 (YP_003137550, YP_003137551), Frankia sp. EuIK1 (AAD17262, AAD17263), Frankia sp. EAN1pec (ABW16212, ABW16211), Frankia alni ACN14a (YP_716936, YP_716935), Heliobacterium chlorum (BAD95756, BAD95757), Klebsiella pneumoniae 342 (YP_002237560, YP_002237559). Methanococcus maripaludis (AAC45517, AAC45518), Paenibacillus terrae HPL-003 (YP_005075595, YP_005075596), Methanococcus maripaludis S2 (NP_987978, NP_987979), Methanococcus vannielii SB (YP_001322586, YP_001322585), Methanothermobacter thermautotrophicus str. Delta H (NP_276678, NP_276679), Paenibacillus sp. Aloe-11 (WP_007429045, WP_007429046), Paenibacillus massiliensis (AAX73208, AAX73209), Rivularia sp. PCC 7116 (YP_007059114, YP_007059113). (JPEG 101 KB) [file 12864_2013_6682_MOESM6_ESM.jpeg]

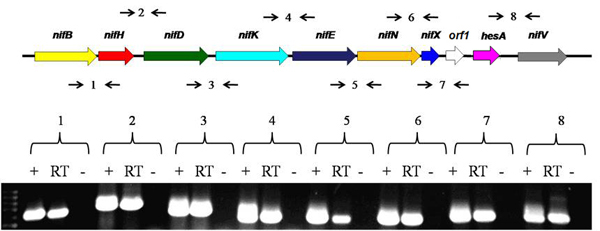

Supplement: Supplementary file 7 — Additional file 7: Figure S7: The ten genes nifB1, nifH1, nifD, nifK, nifE1, nifN1, nifX, orf1, hesA and nifV within the complete nif gene cluster of P. sabinae T27 are organized in an operon as determined by RT-PCR. (A) Outline of the strategy. Primers used and amplified products (numbered) are given below the schematic representation of the genes. (B) Result of RT-PCR reactions with RNA from P. sabinae T27 grown under N2-fixing conditions. The numbering on the top of the gels corresponds to the product numbers drawn schematically in the outline given above. RT, standard RT-PCR reaction; (−), negative control in which no reverse transcriptase was added to the RT reaction; (+), positive control in which genomic DNA was used as template in the RT-PCR. (JPEG 80 KB) [file 12864_2013_6682_MOESM7_ESM.jpeg]

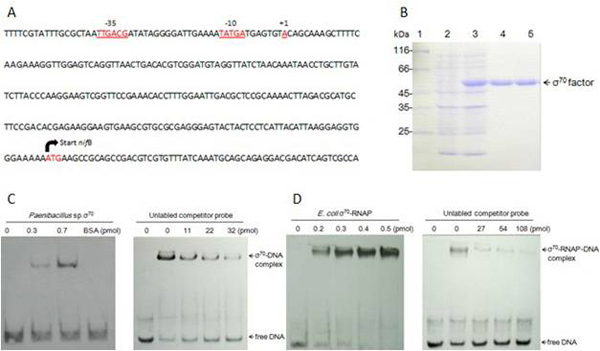

Supplement: Supplementary file 8 — Additional file 8: Figure S8: Characterization of the nif promoter of P. sabinae T27. (A) Nucleotide sequence of the nifB promoter. (B) Overexpression and purification of σ70 from Lane 1: protein marker; lane 2: uninduced protein; lane 3: induced protein; lanes 4: purified σ70 factor. (C) Electrophoretic mobility shift assays (EMSA) demonstrating binding of P. sabinae σ70 to the 45 bp nifB promoter DNA fragment (final concentration 0.03 pmol). The protein concentration is indicated in pmol above each lane (left hand panel). In the right hand panel, the protein concentration was maintained at 2.4 pmol and unlabeled nifB promoter fragment was added as competitor (concentration indicated above each lane). (D) EMSA experiments demonstrating binding of E. coli σ70-RNAP to the 45 bp nifB promoter DNA fragment (final concentration 0.03). The protein concentration is indicated in pmol above each lane (left hand panel). In the right hand panel, the protein concentration was maintained at 0.2 pmol and unlabeled nifB promoter fragment was added as competitor (concentration indicated above each lane). (JPEG 111 KB) [file 12864_2013_6682_MOESM8_ESM.jpeg]

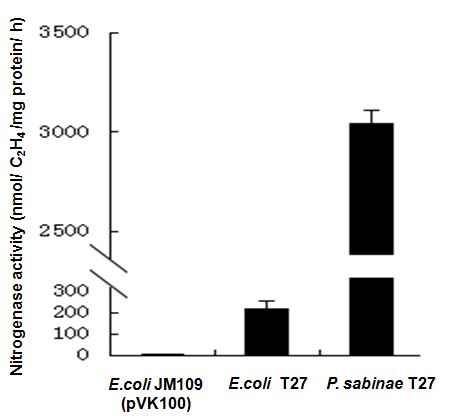

Supplement: Supplementary file 9 — Additional file 9: Figure S9: Nitrogenase activity of E. coli T27, E. coli JM109 and P. sabinae T27. E. coli T27 carrying the complete nif gene cluster from P. sabinae T27. E. coli JM109 carrying the empty vector plasmid pVK100 and P. sabinae T27 are used as negative and positive controls, respectively. Strains were grown anaerobically in nitrogen-deficient conditions. (TIFF 647 KB) [file 12864_2013_6682_MOESM9_ESM.tiff]
