# Supplementary material for: The genome of Paenibacillus sabinae T27 provides insight into evolution, organization and functional elucidation of nif and nif-like genes
Source: BMC Genomics. 2014 Aug 27;15(1):723. doi: 10.1186/1471-2164-15-723 (PMC4246453; doi:10.1186/1471-2164-15-723)
Supplement: Supplementary file 10 — Additional file 10: Table S1: Strains and plasmids used in this research. (DOCX 17 KB) [file 12864_2013_6682_MOESM10_ESM.docx]

Table S1. Strains and plasmids used in this research

| **Strains and plasmids** | **Relevant characteristics** | **References** |
| --- | --- | --- |
| *E. coli* JM109 | *RecA*1 *endA*1 *gyrA*96 *thi hsdR*17 *supE*44 Δ（*lac-proAB*）/ F’ [*traD*36*proA+B+, lacIq lacZ*ΔM15] | Our lab |
| *Paenibacillus sabinae* T27 | Nitrogen-fixing strain | Our lab |
| KP M5a1 | Wild type *K. pneumoniae* | 27 |
| KP 1795 | *K. pneumoniae nifH mutant* | 27 |
| Iɑ423P | *K. pneumoniae nifD* mutant | 26 |
| pVK100 | 23 kb broad-host-range cosmid vector, Km^r^ Tc^r^ | 27 |
