# Supplementary material for: The genome of Paenibacillus sabinae T27 provides insight into evolution, organization and functional elucidation of nif and nif-like genes
Source: BMC Genomics. 2014 Aug 27;15(1):723. doi: 10.1186/1471-2164-15-723 (PMC4246453; doi:10.1186/1471-2164-15-723)
Supplement: Supplementary file 11 — Additional file 11: Table S2: Primers used in this study. (DOCX 24 KB) [file 12864_2013_6682_MOESM11_ESM.docx]

Table S2. Primers used in this study

| **Gene** | **Forward primer (5' - 3')** | **Reverse primer (5' - 3')** | **Location /Target** |
| --- | --- | --- | --- |
| 16S rDNA | 16S-up (AGAGTTTGATCCTGGCTCAGAACGAACGCT) | 16S-down (TACGGCTACCTTGTTACGACTTCACCCC) | RT-PCR (control) |
| Spacer between *nifB* and *nifH* | *nifBH*-F  (GCTGCCCCTGGTCCGCAAG) | *nifBH*-R  (TAGCCATCATTTCGCCGGAAC) | RT-PCR |
| Spacer between *nifH* and *nifD* | *nifHD*-F (TCCCGTAACACCGACCGTGAAGAC) | *nifHD*-R (TTACGCCCGGACGGGATTTAACG) | RT-PCR |
| Spacer between *nifD* and *nifK* | *nifDK*-F (GCATACGAACTGGAAGAACTGGCTC) | *nifDK*-R (GAAGTAAGAGTTACAACCTTGGGAAC) | RT-PCR |
| Spacer between *nifK* and *nifE* | *nifKE*-F (CGGTTTATGTTGGCAAAGACTTGTG) | *nifKE*-R (TCCATTGGATCATCATCTCCTTCGC) | RT-PCR |
| Spacer between *nifE* and *nifN* | *nifEN*-F (CCGGATTATTAAGACGGTTAGAGAG) | *nifEN*-R  (ATGCTTGGACAGTACCGCATTC) | RT-PCR |
| Spacer between *nifN* and *nifX* | *nifNX*-F  (CGTATATGAATGTCTCCGTTGGC) | *nifNX*-R (GTTCATTCGCGGGTGCGATTTCG) | RT-PCR |
| Spacer of *nifX* and *hesA* | *nifXhesA*-F (CAAAGCTCTGCGGAAATCAACGTCG) | *nifXhesA*-R (CAGAATCTGCCGGTTCAAGTCCGGC) | RT-PCR |
| Spacer between *nifX* and *hesA* | *hesAnifV*-F (GGGATTTCCAGTTTTGGGCGCAACC) | *hesAnifV*-R (TTAAGCCTTCGGCGGGCGTTAGTCC) | RT-PCR |
| The complete *nif* cluster of *P. sabinae* T27 | T-up (TCCTCGAGGCCGGAGAACCATAACG) | T-down (CTCTCGAGATAGGGATAACCATCCGCC) | For expression of *nif* cluster |
|  | T27-GSP1 (CATGCTTTCTGACTCTGGCAAAC) | T27-GSP2 (GCTGCATCAATTGTGCCGCCACACCTT) | 5' RACE |
|  | Random primer mix | T27-GSP3 (AAGGGTGGCGACTGATGTCGTC) | 5' RACE |
| *rpoD* | A-F (CCGGAATTCAATGGCGAACGATCAGCATACC) | A-R  (ACGCGTCGACCCTTTAAAGGCTGATTTG) | *P. sabina*e T27 σ^70^ |
|  | T27promoter+ (GCTAATTGACGATATAGGGGATTGAAAATATGATGAGTGTACAGC) | T27promoter- (GCTGTACACTCATCATATTTTCAATCCCCTATATCGTCAATTAGC) | EMSA |
| *nifD* gene | D1-F  (CACAGTCATATCCATGTCCTTGG) | D1-R  (GTCAAATGCACTCCTGGGATTA) | q-RT-PCR |
| *nifD*-like1 gene | D2-F  (GAACGAAATCAGACAACGGCAAC) | D2-F  (GGATTGCGCCTATCACCTCG) | q-RT-PCR |
| *nifD*-like2 gene | D3-F  (CATTAAACATAAAGTGCCGCCGG) | D3-R  (CTGGGTTTGG GAGAAGGTGCG) | q-RT-PCR |
| *nifD*-like3 gene | D4-F  (GAATTTAACC GAGGTTCCGGTGC) | D4-R  (GTTCCCTGAGCTGCAGCCCATG) | q-RT-PCR |
| *nifD*-like4 gene | D5-F  (GACTGAAATCCATTGTAGGGTATC) | D5-R  (CCGTGATCGTGGACAGATAC) | q-RT-PCR |
| *nifD*-like5 gene | D6-F  (CTTTGACGCGACCCAGCATG) | D6-R  (GATCGGAGACGGATGCCATATC) | q-RT-PCR |
| *nifK* gene | K1-F (CGATGCATATGATGACGGTCAAAG) | K1-R  (GGTTTATGTTGGCAAAGACTTGTG) | q-RT-PCR |
| *nifK*-like1 gene | K2-F  (GAGAGACAAC GGCGGGAAAC) | K2-R  (GTTGAAGGAAGACCGAGGCAC) | q-RT-PCR |
| *nifK*-like2 gene | K3-F  (GTCGGGGATCACGGAACTTCGC) | K3-R  (CCAGTCGCTC CAGATAAGGG TAG) | q-RT-PCR |
| *nifK*-like3 gene | K4-F  (GAAGATCAGG CAGACCAATC C) | K4-R  (GTAGCCGCAGTTTAAGATCAGC) | q-RT-PCR |
| *nifK*-like4 gene | K5-F  (CTGCGTAGCGAAGCTGTGGAC) | K5-R  (AGTGTGCACACATGTCTAATTTGC) | q-RT-PCR |
| *nifK*-like5 gene | K6-F  (CTGTTACCAAAAATGAGCACG) | K6-R  (GAATGGATAC GACGCTGTAC AG) | q-RT-PCR |
| *nifH1* gene | H1-F  (AATTAGTGAC CAGCAGCTTC) | H1-R  (AGCCAACGAA TACAAACAAC) | q-RT-PCR |
| *nifH2* gene | H2-F  (GACGACCACATCCAACATCAGTG) | H2-R  (CAGCTTCACATTGGCGCTGTC) | q-RT-PCR |
| *nifH*  gene | H3-F  (GCAATCTACGGCAAAGGGGGG) | H3-R  (CGAATCGCTC TTTGGGTCGC) | q-RT-PCR |
| *nifH*-like1 gene | H4-F  (AGCCTAGGGCTTCTCTTCGC) | H4-R  (GCAGCAGCCA ACGCCTAAGC) | q-RT-PCR |
| *nifH*-like2 gene | H5-F (GAGAACCCGATTATGACTAAAAAAC) | H5-R  (CTTTGGTGTT CAGAATCAGG C) | q-RT-PCR |
| *K. pneumoniae* *nifH* promoter for overlap PCR with *K. pneumoniae nifD* | A1 (GTTGTCGACTCGAAGGTGAGAGGCATCTT) | A2(K.P) (TAATTGTCCTGGTGACTTCTTCTGTTGTTG ) | Complementation |
| *K. pneumoniae* *nifH* promoter for overlap PCR with *P. sabinae* T27 *nifD* | A1(GTTGTCGACTCGAAGGTGAGAGGCATCTT) | A2(*nifD1*) CCAGTCCCATGGTGACTTCTCCTGTTGTTG) | Complementation |
| *K. pneumoniae* *nifH* promoter for overlap PCR with *P. sabinae* T27 *nifD*-like1 | A1(GTTGTCGACTCGAAGGTGAGAGGCATCTT) | A2(*nifD2*) TTTCGTTCATGGTGACTTCTCCTGTTGTTG) | Complementation |
| *K. pneumoniae* *nifH* promoter for overlap PCR with *P. sabinae* T27 *nifD*-like2 | A1 (GTTGTCGACTCGAAGGTGAGAGGCATCTT) | A2(*nifD3*) (TGTAATCCATGGTGACTTCTCCTGTTGTTG) | Complementation |
| *K. pneumoniae* *nifH* promoter for overlap PCR with *P. sabinae* T27 *nifD*-like3 | A1 (GTTGTCGACTCGAAGGTGAGAGGCATCTT) | A2(*nifD4*) (CTTTCGGCATGGTGACTTCTCCTGTTGTTG) | Complementation |
| *K. pneumoniae* *nifH* promoter for overlap PCR with *P. sabinae* T27 *nifD*-like4 | A1 (GTTGTCGACTCGAAGGTGAGAGGCATCTT) | A2(*nifD5*) (AATGGCCAAGGTGACTTCTCCTGTTGTTG) | Complementation |
| *K. pneumoniae* *nifH* promoter for overlap PCR with *P. sabinae* T27 *nifD*-like5 | A1 (GTTGTCGACTCGAAGGTGAGAGGCATCTT) | A2(*nifD6*) (TCTTGCCCATGGTGACTTCTCCTGTTGTTG) | Complementation |
| *K. pneumoniae* *nifH* promoter for overlap PCR with *P. sabinae* T27 *nifH*-like1 | A1 (GTTGTCGACTCGAAGGTGAGAGGCATCTT) | A2(*nifH*-like1) (TTTAGCCATGGTGACTTCTCCTGTTGTTG) | Complementation |
| *K. pneumoniae* *nifH* promoter for overlap PCR with *P. sabinae* T27 *nifH*-like2 | A1 (GTTGTCGACTCGAAGGTGAGAGGCATCTT) | A2 (*nifH*-like2)(CTGCACTCCGTGACTTCTCCTGTTGTTG) | Complementation |
| *P. sabinae* T27 *nifD* for overlap PCR with *K.pneumoniae nifH* promoter | *nifD1*-F (AGAAGTCACCATGGGACTGGATATTGAGGC) | *nifD1*-R (AACCTCGAGTGATAACAACCGCTTTAC) | Complementation |
| *P. sabinae* T27 *nifD*-like1 for overlap PCR with *K. pneumoniae nifH* promoter | *nifD2*-F (AGAAGTCACCATGAACGAAATCAGACAAC) | *nifD2*-R (TTGCTCGAGTCCTTGAGACCTTTCAGATTTC) | Complementation |
| *P. sabinae* T27 *nifD*-like2 for overlap PCR with *K. pneumoniae nifH* promoter | *nifD3*-F (AGAAGTCACCATGGATTACATTAAACATAAAGTGCC) | *nifD3*-R (TTGCTCGAGCATTCACCCTTCCTTTTACTTATTC) | Complementation |
| *P. sabinae* T27 *nifD*-like3 for overlap PCR with *K. pneumoniae nifH* promoter | *nifD4*-F (AGAAGTCACCATGCCGAAAGTCAACTTGAATTTAAC) | *nifD4*-R (AACCTCAGATATGGAATGGAAGCAAAGAC) | Complementation |
| *P. sabinae* T27 *nifD*-like4 for overlap PCR with *K. pneumoniae nifH* promoter | *nifD5*-F (AGAAGTCACCTTGGCCATTCATTTAAATCTCTC) | *nifD5*-R (TTCGTCGACAAGGTATTGGTGATCACTTTGCGCAG) | Complementation |
| *P. sabinae* T27 *nifD*-like5 for overlap PCR with *K. pneumoniae nifH* promoter | *nifD6*-F (AGAAGTCACCATGGGCAAGATCAATTTGAG) | *nifD6*-R (TTGGTCGACGCGAACTTGATTGATTGAATTGG) | Complementation |
| *K. pneumoniae nifD* for overlap PCR with *K. pneumoniae nifH* promoter | *nifD*(KP)-F (AGAAGTCACCAGGACAATTATGATGACCAAC) | *nifD*(KP)-R (TTGAAGCTTAATCGTTTGGCTCATGGTGTTC) | Complementation |
| *P. sabinae* T27 *nifH*-like1 for overlap PCR with *K. pneumoniae nifH* promoter | *nifH*-like1-F (AGAAGTCACCATGGCTAAAAGGACCAAGC) | *nifH*-like1-R (CTGAAGCTTCTCTGCGTTTTTCCCTCGTC) | Complementation |
| *P. sabinae* T27 *nifH*-like2 for overlap PCR with *K. pneumoniae nifH* promoter | *nifH*-like2-F (AGAAGTCACGGAGTGCAGTTTAATGGCT) | *nifH*-like2-R (TTGAAGCTTCGAGATGGAGTTCTGGTC) | Complementation |
